# Supplementary figures and images for: Comparative analysis reveals the species-specific genetic determinants of ACE2 required for SARS-CoV-2 entry
Source: PLoS Pathog. 2021 Mar 24;17(3):e1009392. doi: 10.1371/journal.ppat.1009392 (PMC7990223; doi:10.1371/journal.ppat.1009392)

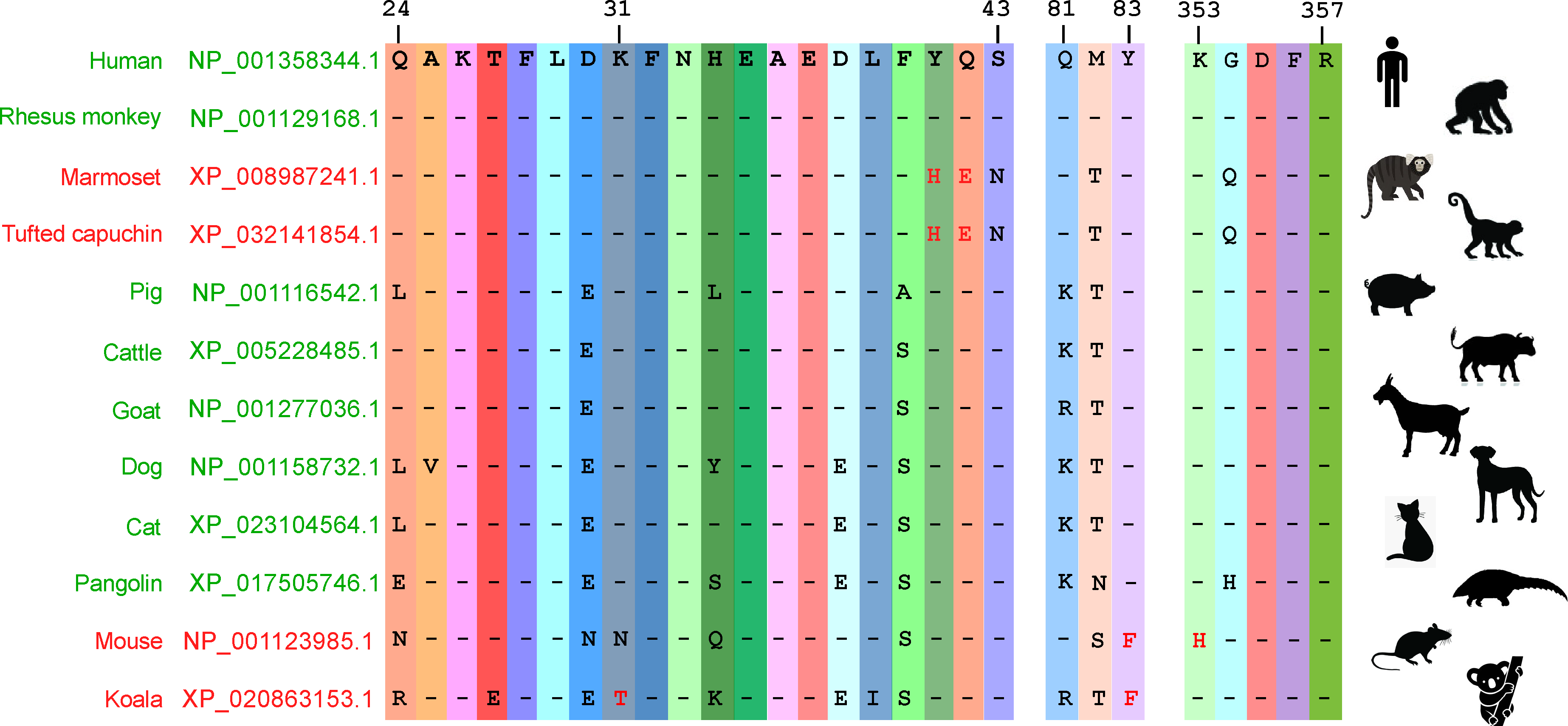

Supplement: S1 Fig — Multiple ACE2 orthologs were retrieved from NCBI database and the counterpart residues of ACE2 orthologs at interfaces of human ACE2 with SARS-CoV-2 spike were aligned using MEGA. ACE2 orthologs highlighted in green were susceptible to SARS-CoV-2, and species highlighted in red were resistant to SARS-CoV-2. The restrictive residues of koala or mouse ACE2 are highlighted in red. (TIF) [file ppat.1009392.s001.tif]

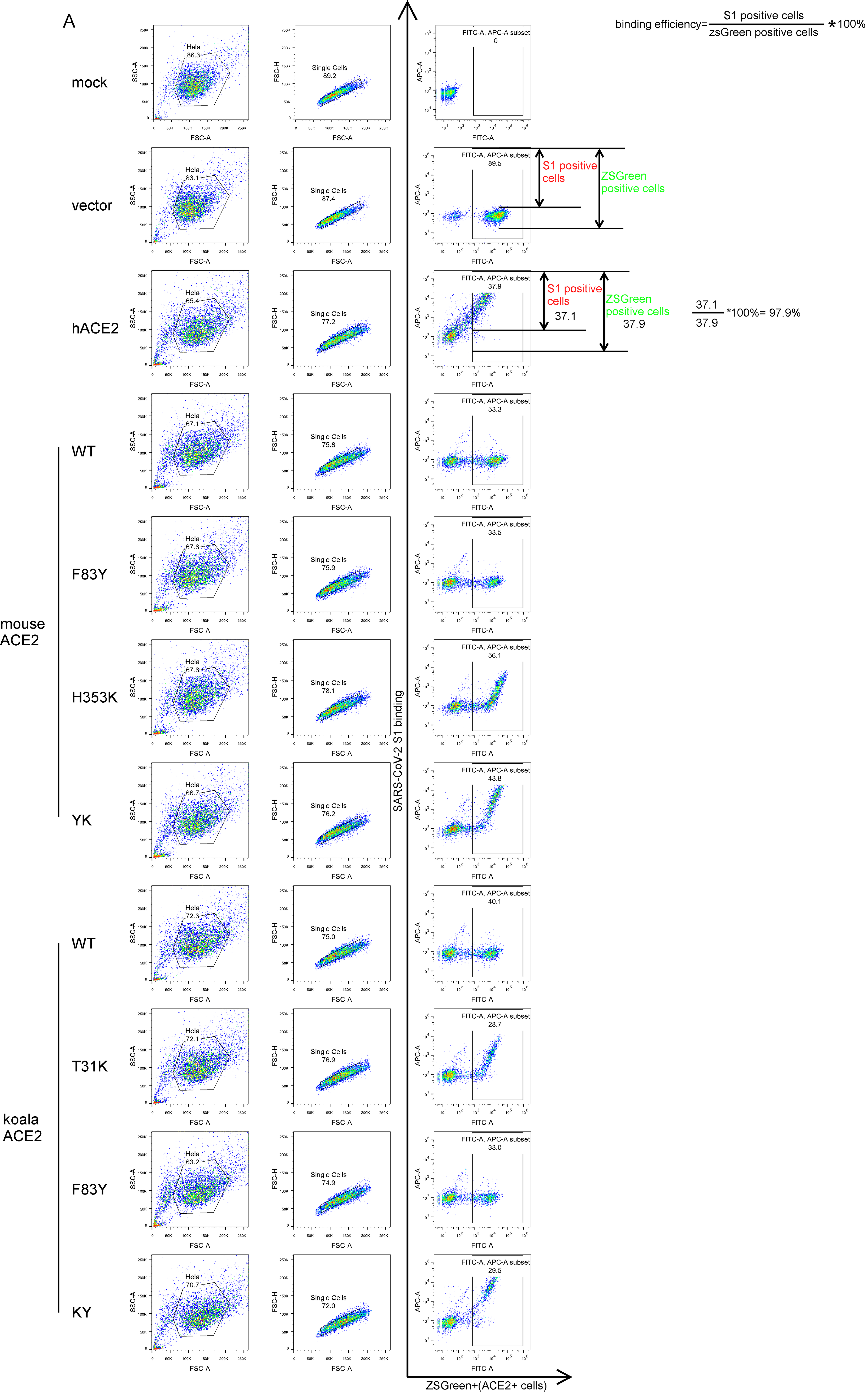

Supplement: S2 Fig — (A-B) Main cell population was identified and gated on Forward and Side Scatter. Single cells were further gated on FSC-A and FSC-H. The gated cells were plotted by FITC-A (zsGreen, as the ACE2 expressing population) and APC-A (S1-Fc bound population). The FITC-A positive cell population was plotted as a histogram to show the S1-Fc positive population as Fig 2B. The binding efficiency was defined as the percent of S1-Fc binding cells among the zsGreen positive cells. Shown are FACS plots representative of those that have been used for the calculations of binding efficiencies of ACE2 variants with S1-Fc. This experiment was independently repeated three times with similar results. (ZIP) [file ppat.1009392.s002.zip › S2_Fig.zip/S2A_Fig.tif]

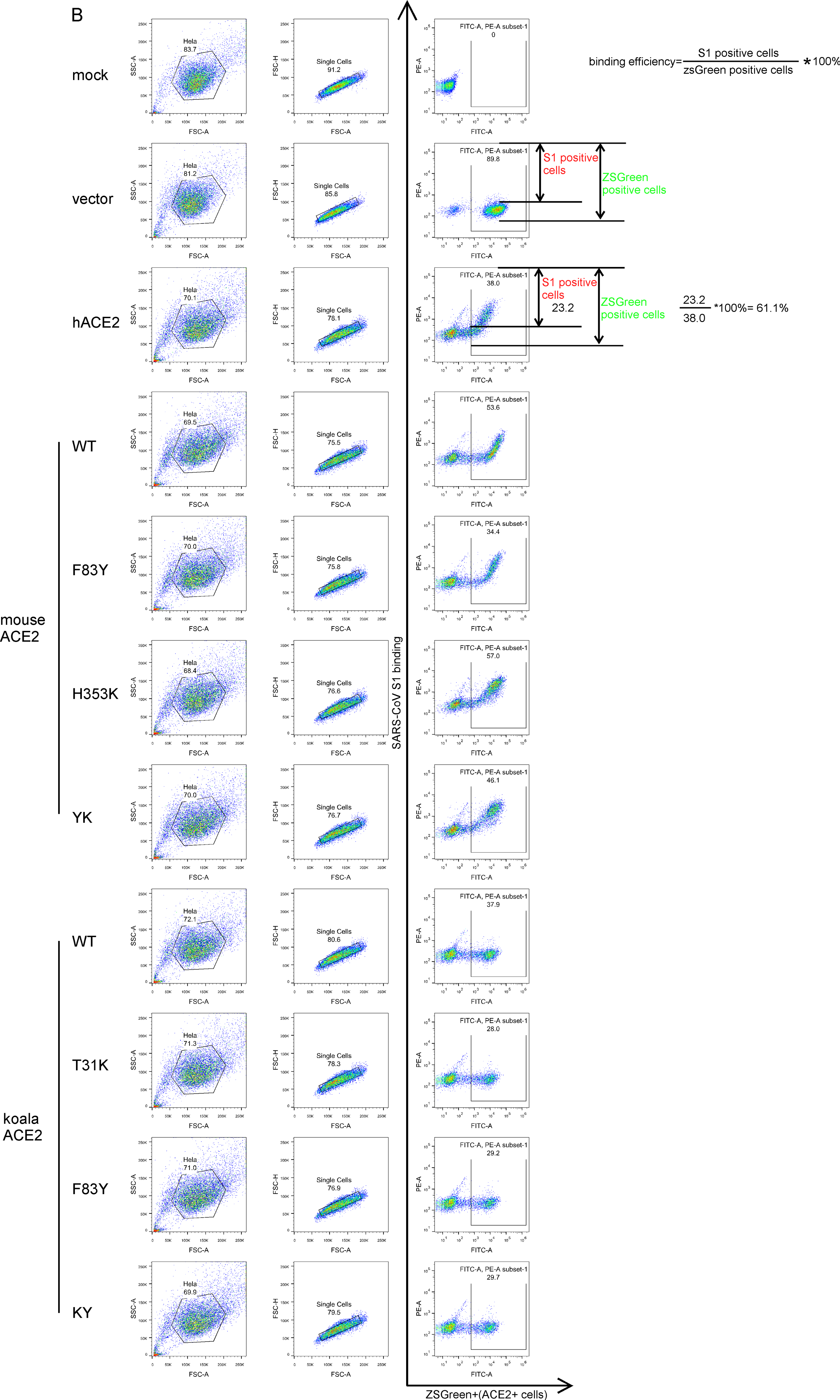

Supplement: S2 Fig — (A-B) Main cell population was identified and gated on Forward and Side Scatter. Single cells were further gated on FSC-A and FSC-H. The gated cells were plotted by FITC-A (zsGreen, as the ACE2 expressing population) and APC-A (S1-Fc bound population). The FITC-A positive cell population was plotted as a histogram to show the S1-Fc positive population as Fig 2B. The binding efficiency was defined as the percent of S1-Fc binding cells among the zsGreen positive cells. Shown are FACS plots representative of those that have been used for the calculations of binding efficiencies of ACE2 variants with S1-Fc. This experiment was independently repeated three times with similar results. (ZIP) [file ppat.1009392.s002.zip › S2_Fig.zip/S2B_Fig.tif]

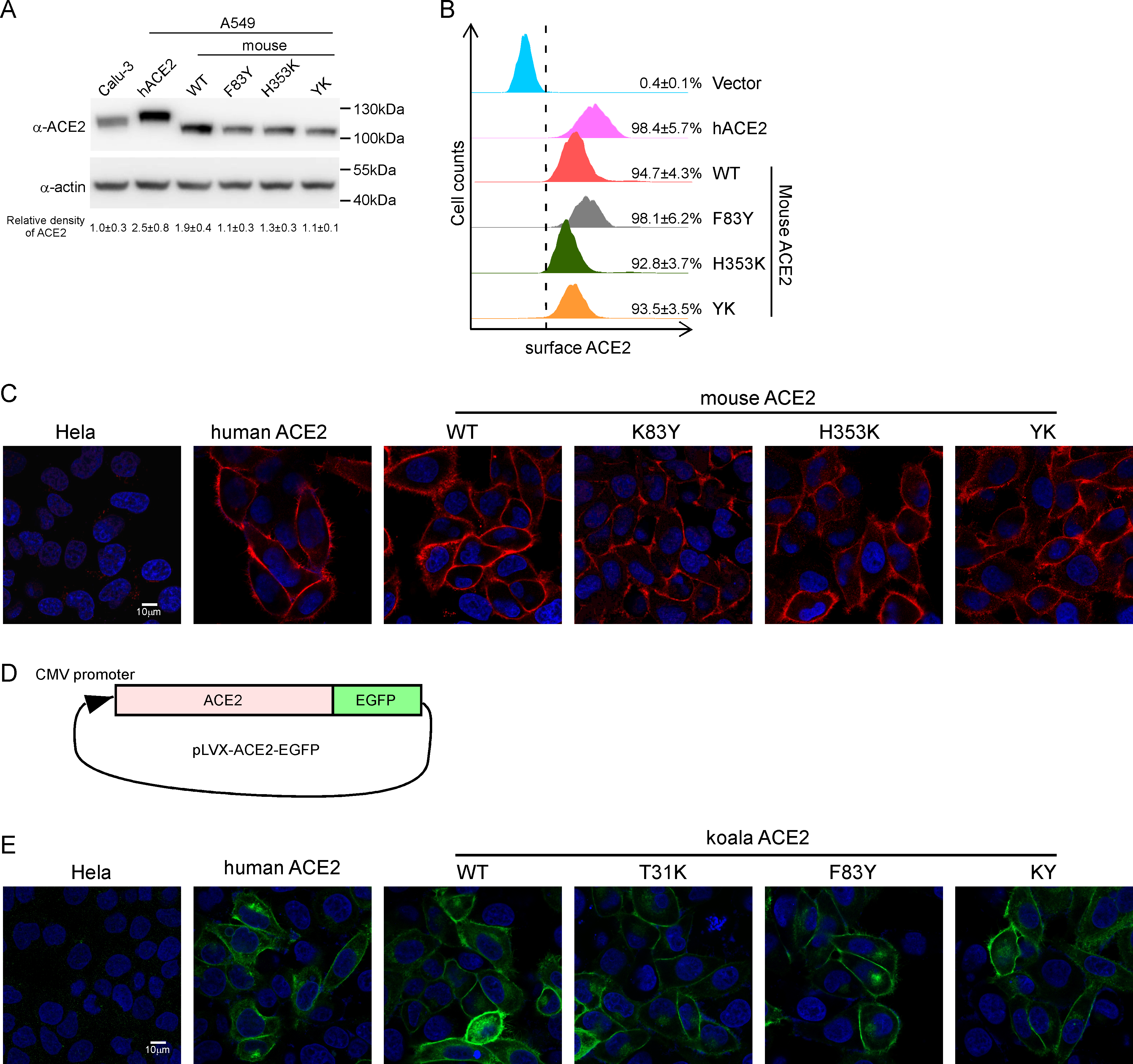

Supplement: S3 Fig — (A) Western blotting assay was performed to detect expression of endogenous ACE2 in Calu-3 cells and ACE2 variants in A549 cells transduced by lentiviruses. (B) A549 cells transduced with lentiviruses (pLVX-IRES-zsGreen) expressing ACE2 variants were incubated with rabbit polyclonal antibody (Sino Biological Inc. China, Cat: 10108-T24) against ACE2. The cells were washed and then stained with 2μg/mL goat anti-rabbit IgG (H+L) conjugated with Alexa Fluor 568 for flow cytometry analysis. The cell surface ACE2 was calculated as the percent of Alex Fluor 568 positive cells among the zsGreen positive cells. This experiment was repeated three with similar result. (C) A549 cells transduced with lentiviruses (pLVX-IRES-zsGreen) expressing ACE2 variants were incubated with rabbit polyclonal antibody (Sino Biological Inc. China, Cat: 10108-T24) against ACE2. The cells were washed and then stained with 2μg/mL goat anti-rabbit IgG (H+L) conjugated with Alexa Fluor 568 and DAPI (1μg/ml). The cell images were captured with a Zeiss LSM 880 Confocal Microscope. ACE2 on cell surface was shown in the merge images processed by ZEN3.2 software. This experiment was independently repeated three with similar result and the representative images were shown. (D) Human, koala ACE2 or its variants cDNA was cloned as a carboxyl terminus fusion with EGFP. (E) A549 cells were transduced with lentivirus expressing ACE2 variant-EGFP proteins in (D) and cells were collected, washed and counterstained with DAPI (1μg/ml). The cell images were captured with a Zeiss LSM 880 Confocal Microscope. ACE2 on cell surface was shown in the merge images processed by ZEN3.2 software. This experiment was independently repeated three times with similar result and the representative images were shown. (TIF) [file ppat.1009392.s003.tif]

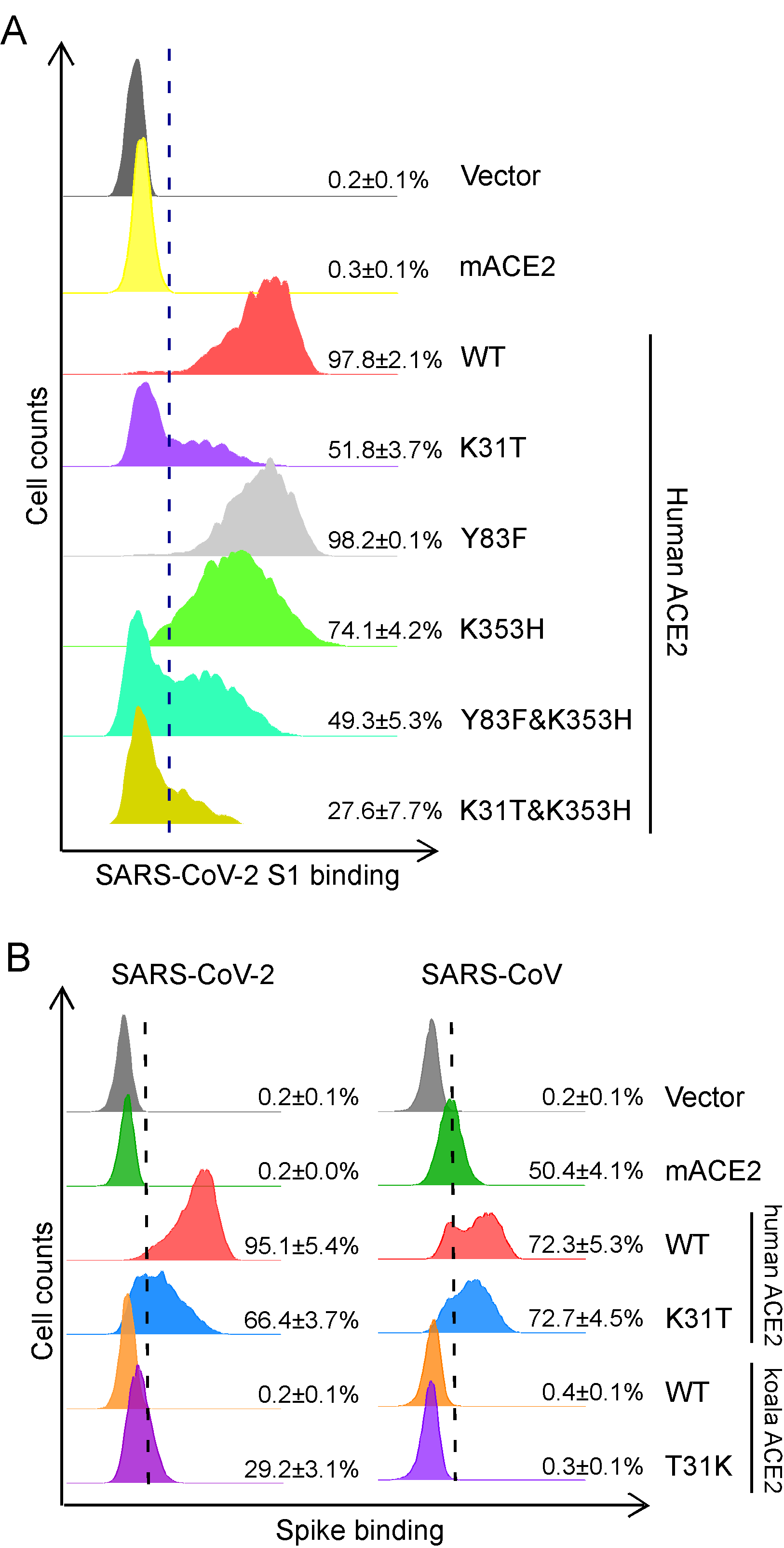

Supplement: S4 Fig — (A) A549 cells were transduced with ACE2 orthologs or human ACE2 mutants as indicated, incubated with the recombinant S1 domain of the SARS-CoV-2 or SARS-CoV spike protein C-terminally fused with Fc, and then stained with goat anti-human IgG (H + L) conjugated to Alexa Fluor 647 for flow cytometry analysis. (B) The A549 cells transduced with ACE2 orthologs and their variants as indicated, and the cells were incubated with full-length spike protein of SARS-CoV-2 (SinoBiological, 40589-V08B1) or SARS-CoV (SinoBiological, 40634-V08B) C-terminally fused with His tag, and then stained with Anti-HIS-PE (Miltenyi Biotec#130-120-787). Values in (A) and (B) are expressed as the percent of cells positive for S1-Fc among the ACE2-expressing cells (zsGreen1+ cells) and shown as the means ± SD from 3 biological replicates. This experiments were independently performed three times with similar results. (TIF) [file ppat.1009392.s004.tif]
